# A Participatory Sensing Study to Understand the Problems Older Adults Faced in Developing Medication-Taking Habits

**DOI:** 10.3390/healthcare10071238

**Published:** 2022-07-02

**Authors:** Maribel Valenzuela-Beltrán, Ángel G. Andrade, Katarzyna Stawarz, Marcela D. Rodríguez

**Affiliations:** 1 Facultad de Ingeniería, Universidad Autónoma de Baja California (UABC), Mexicali 21100, Mexico; maribel.valenzuela@uabc.edu.mx (M.V.-B.); aandrade@uabc.edu.mx (Á.G.A.); 2 School of Computer Science and Informatics, Cardiff University, Cardiff CF24 4AG, UK; stawarzk@cardiff.ac.uk

**Keywords:** older adults, medication-taking behaviors, medication adherence, medication consistency

## Abstract

Past research has demonstrated that older adults tend to use daily activities as cues to remember to take medications. However, they may still experience medication non-adherence because they did not select adequate contextual cues or face situations that interfere with their medication routines. This work addresses two research questions: (1) How does the association that older adults establish between their daily routines and their medication taking enable them to perform it consistently? (2) What problems do they face in associating daily routines with medication taking? For 30 days, using a mixed-methods approach, we collected quantitative and qualitative data from four participants aged 70–73 years old about their medication taking. We confirm that older adults who matched their medication regimens to their habitual routines obtained better results on time-based consistency measures. The main constraints for using daily routines as contextual cues were the insertion of medication taking into broad daily routines, the association of multiple daily routines with medication taking, the lack of strict daily routines, and the disruption of daily routines. We argue that the strategies proposed by the literature for forming medication-taking habits should support their formulation by measuring patients’ dosage patterns and generating logs of their daily activities.

## 1. Introduction

Worldwide, medication non-adherence is a significant challenge [1]. Medication adherence is the degree to which patients’ medication-taking behaviors correspond to the health providers’ recommended pharmacotherapy, comprising the prescribed dose and interval of their medication regimen [2,3]. Medication non-adherence is comprised of multiple behaviors that may occur across the three phases of medication taking: the initiation of the treatment (e.g., not filling the initial prescription or not taking the first doses), the implementation of the prescribed regimen (e.g., taking doses at the wrong time, missing doses), and persistence (e.g., early discontinuation of the pharmacotherapy) [2,4].

Different studies have demonstrated that poor adherence to prescribed drug treatments is one of the leading causes of illness and treatment failure, which increases emergency room visits and re-hospitalizations [5]. Specifically, the possibility of medication-related readmissions is quite high in older adults [5,6]. Thus, non-adherence has become a medication-related problem contributing to the total global health expenditure, estimated at USD 42 billion annually [7]. Specifically, the non-adherence problem predominates in approximately 50% of the population aged 65 and older [8,9]. Studies suggest that the behavioral strategies benefit the medication adherence of older adults more than that of the cognitively oriented ones [10,11]. The former includes remembering medication, monitoring the taken pills, giving feedback, and forming habits. In contrast, the cognitive ones focus on changing knowledge and beliefs [10,11]. Moreover, among behavioral strategies, those aimed at developing habits benefit adherence to medication the most [11]. 

Habits form when the behaviour is consistently repeated in the presence of contextual cues [12]. This process can be instigated by goals [13] or by contextual cues [12]. In the context of medication taking, both play a role. First, an intention to take a specific action must be formulated [14,15,16,17,18,19], e.g., a person should take one pill of metformin after breakfast. Next, to form the habit, the intention needs to turn into action [14,15], and that action needs to be repeated—the person starts taking one pill of metformin after breakfast and then continues to do so consistently for several weeks. The importance of intentions decreases as the behavior is repeated, and with time, the activity can be automatically completed without reconsidering its purpose [19]. Creating salient cues contributes to the activity consistency and reduces behavioral complexity, while giving extrinsic rewards might hinder the habit-formation process [12]. Finally, the behavior must be maintained to become a habit and bring long-lasting change [12,14,15,16,17,18,19]. Thus, behaviorists consider that a habit can be acquired through the repetition of behavior in the presence of contextual cues [12]. In this approach, contextual cues reflect features of the environment in which the planned activity typically occurs [20,21]. Therefore, they act as reminders, prompting people to act consciously [20,21]. A cue refers to the place, time, used objects, and daily routines associated with the environment where the medication taking needs to be performed [22,23,24]. The expected benefit of using cue-based strategies is that the probability of discontinuing the treatment is reduced when the medication taking acquires automaticity. It helps to overcome forgetfulness (unintentional non-adherence) and prolong persistence [24]. However, previous research has determined that older adults continue having trouble with medication tasks that have become habitual since they need compensatory memory support to help them cope with situations that interfere with their routine [25]. A clinical trial demonstrated that training older adults to support the formation of medication-taking habits improves their medication consistency; however, it should be noted that the improvements were sustained only for as long as the researchers maintained frequent contact with the participants [26]. The studies conducted so far have been limited to identifying the daily routines most used as contextual cues of medication by older adults [27,28], understanding how these are used as compensatory memory strategies [25], and understanding the factors that influence the selection of daily routines to be used as contextual cues [20]. As these studies have collected data through self-report techniques such as surveys and interviews conducted in a single session, they provide limited evidence about the effect of the cue-based strategies selected by older adults on the consistency of their medication-taking behaviors. Furthermore, the validity of self-reports of behaviors has been questioned since they do not provide objective data to measure and explain the operationalization of habitual behaviors [29]. Therefore, it remains unclear how the cue-based strategies established by older adults impact their medication-taking behaviors. As such, the research questions (RQs) of this work are: 

RQ1: How does the association that older adults establish between their daily routines and their medication taking enable them to perform it consistently?

RQ2: What problems do older adults face in associating their daily routines with their medication taking?

## 2. Method

### 2.1. Study Design and Setting

To address the RQs, we conducted a field study to collect data from four older adults. They reported their medication-taking behaviors and daily routines using a data-gathering platform. As a result, we obtained quantitative and qualitative evidence about their medication consistency and the problems they faced in developing medication-taking habits.

This study is the first stage of a larger project that aims to develop interactive technology to support older adults to form medication-taking habits. For developing our project, we follow the usability specification and evaluation framework for health information technology [30]. According to it, an initial and simple study type comprising field or lab studies can be conducted to understand the task to support and thus discover essential system requirements. In this sense, our study aims to understand the task as stated in the research questions, for which we used the contextual inquiry technique [31]. Contextual inquiry is a field data-gathering technique used in the user-centered system design process [32]. It is a combination of in situ interviews and observations, for which we used participatory sensing technology. Combining these methods ensures the capture of real users’ practices and daily activities instead of only using interviews, for which four to six participants are required to reach data saturation [31,33], especially for longer studies. As users are the center of our project’s methodological approach (User-Centered Design [32]), we included only older adults in this study.

### 2.2. Recruitment of Participants

We recruited participants by posting study posters in the lead author’s department halls. The inclusion criteria included: being at least 65 years old, taking prescription medications, and receiving no help taking them. We obtained informed consent from the recruited participants, both verbal and written, which included full disclosure of the purpose of the investigation. The older adults received MXN 500 (the equivalent to USD 25, currently) per week during the study duration. 

### 2.3. Measurements

There is no gold standard method for measuring medication adherence, but behavior-change scientists must select the nonadherence behavior(s) relevant for their research and a suitable measurement approach [4,8]. We determined the study variables based on habit and medication adherence definitions. It includes complying with the appropriate time interval between doses, e.g., to take a pill every 12 h apart [34]. On the other hand, studies on habit formation recognize the importance of measuring cue consistency [35,36] and, similarly to our work, have analyzed the consistency of behavior over time, without determining the influence of the contextual cue on it [36].

We established the following measurements to assess the consistency of prescribed medication regimens (RQ1):

- *Time-based consistency.* We measured two relevant aspects:
  (i) Variance (*S*^2^) of the time of day, i.e., how the time of day of medication intake varied from one day to another; and
  (ii) Variance (*S*^2^) of time interval, i.e., whether the medication was taken leaving the appropriate time apart between episodes of the same group of medications, as per the prescribed frequency. Thus, lower variances would be better because the doses would be more evenly spread out, which would ensure that the level of medication in the individual’s system remains constant every day.
- *Cue consistency.* The cue consistency was estimated as a percentage of the number of times the activity set as a contextual cue by the older adults was reported as triggering the medication episode, divided by the number of days of the study duration (30 days).

### 2.4. Data Collection 

We carried out two stages of data collection. The first consisted of an initial semi-structured interview to find out the general characteristics of their medication behaviors. The second aimed to delve into these in more detail to collect quantitative and qualitative data.

#### 2.4.1. Initial Semi-Structured Interview

An initial 40–60 min semi-structured interview enabled us to confirm whether the contacted older adults were appropriate for the study and to gather information about how their medication regimens fit into their daily routine. We asked them to show us the written prescription given by their doctors and to describe it, indicating how they performed a typical medication routine daily. For each drug, they mentioned the dosage frequency, where they take it, the tools used to medicate, and the activities carried out before and after taking it. Then, we asked them which activity they usually associated with the intake of each medication episode. The reported activity was set as the contextual cue. Afterward, they responded to questions such as: How often do you forget to take your medication? How do you realize that you forgot to take your medicines? What are the causes of failing to take medication? Finally, we asked them to describe the daily routines performed on a typical day, including their medication activities. We audio-recorded the interviews and then transcribed them to aid in the analysis. Finally, we also found out if they had the necessary technical skills to participate in the following study stage and if any of their family members could assist them, if required. 

#### 2.4.2. Participatory Sensing Data Collection

**Figure 1:**
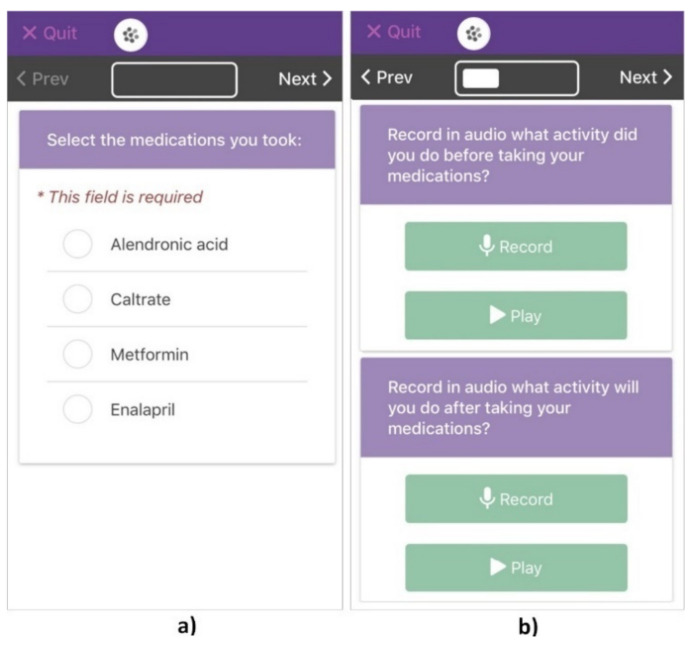
The tablet-based system implemented with EpiCollect5 [37] presents: (**a**) the list of a participant’s medications to be checked if they were taken, and (**b**) the options to audio-record the activities conducted before and after the medication episode.

We collected data for 30 days about the measures related to the consistency of the participants’ medication-taking behaviors. To this end, we implemented a tablet-based system that we provided to the participants. We attached NFC (Near Field Communication) tags to their pill containers to obtain information about each medication episode. The participants were asked to bring the containers close to the tablet immediately after performing a medication episode. This action launched a survey implemented with the EpiCollect5 system [37], shown in Figure 1. It asked the participants to confirm the medicines taken as part of the medication episode (see Figure 1a) and then describe the activities conducted before and after through audio messages (see Figure 1b). Thus, the tablet-based system recorded each set of medication episode-related data, including the name of the medications taken, the current time, and the associated activities. Weekly, we interviewed the participants to clarify any inconsistencies recorded. For instance, we asked about the causes of the changes in the time interval between medication-taking episodes or the reasons for not associating a medication with the activity set as the contextual cue. All interviews were audio-recorded. The participants with the skills to use the tablet-based system were required to register all their medication episodes. Those who needed assistance were asked to record data only on the medications taken when a family member was home to assist them.

### 2.5. Data Analysis 

We organized drugs that should be taken at the same time interval (e.g., every 12 h) into groups (G). In addition, we specified whether each group was taken in the morning (AM) or evening/night (PM) episodes. We estimated the *time interval* variance for each group and the *time-of-day* variance for groups taken as part of the same medication episode. Based on our results, we considered that those participants with variances higher than 1.0 (1 h) had the most inconsistent medication-taking behaviors. We used the qualitative data obtained from the weekly interviews and audio messages to determine the causes of participants’ behaviors. 

We conducted a thematic analysis of the data obtained from the weekly interviews and audio messages, following the steps outlined by Baun & Clarke [38]. In accordance with the method, the first author transcribed the data verbatim. Using an inductive approach, she identified data (i.e., fragments from the transcripts) evidencing situations that impeded using contextual cues and taking the medication on time. She categorized the data based on similar problem situations and proposed the themes that framed each category. The first and last authors met to review and discuss the proposed data categorization and the potential themes. The above process was carried out iteratively to modify and validate the findings. All of the authors reviewed the final version of the themes to provide additional feedback and refine them. 

## 3. Results

### 3.1. Setting and Participants

As shown in Table 1, we conducted the study with four older adults (three women and one man) aged 70–73 years old in Mexicali, Mexico. They were primarily low-income and affiliated with the Mexican Institute of Social Security (IMSS), the largest national medical institution. They reported attending an IMSS clinic at least monthly for follow-up consultations and receiving an updated prescription to get their medications from the clinic’s pharmacy. 

### 3.2. Medication Regimens and Routines 

**Figure 2:**
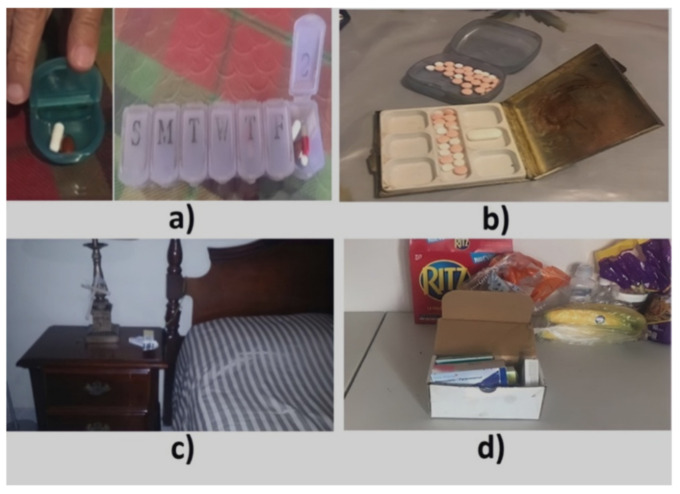
Locations and pill containers used by (**a**) S1, (**b**) S2, (**c**) S3, and (**d**) S4.

**Figure 3:**
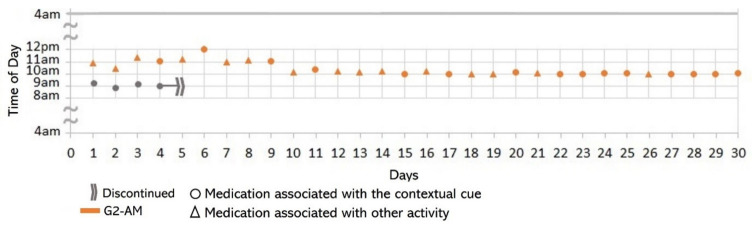
Medication behavior of S1 shows the time of day in which the group of medication (G2) was taken daily and whether G2 was associated with the activity set as the contextual cue.

**Figure 4:**
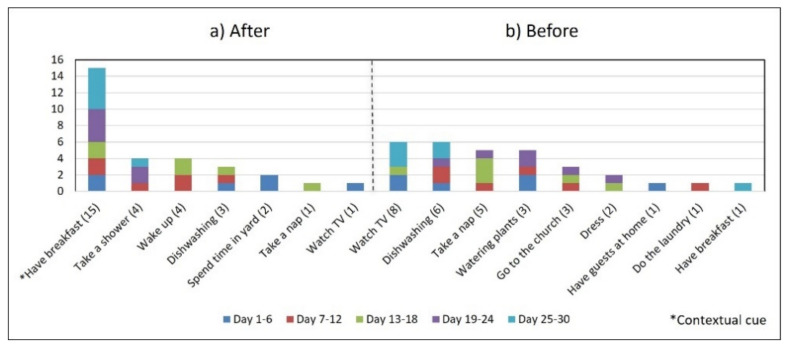
The number of days S1 performed the medication episode (G2-AM) after and before the reported activities.

**Figure 5:**
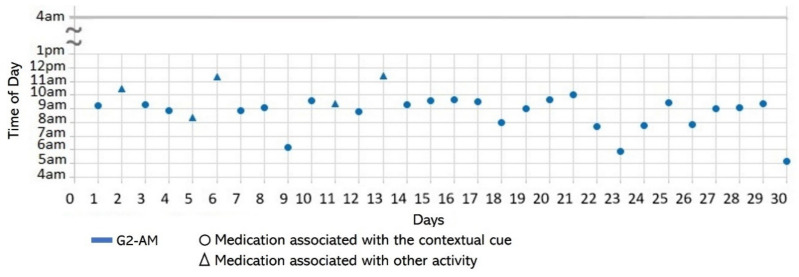
Medication behavior of S2 shows the time of day in which the group of medication (G1) was taken daily and whether it was associated with the activity set as the contextual cue.

**Figure 6:**
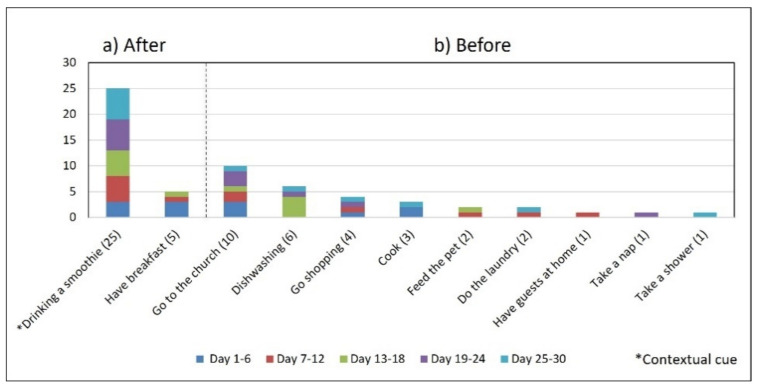
The number of days S2 performed the medication episode (G1-AM) after and before the reported activities.

**Figure 7:**
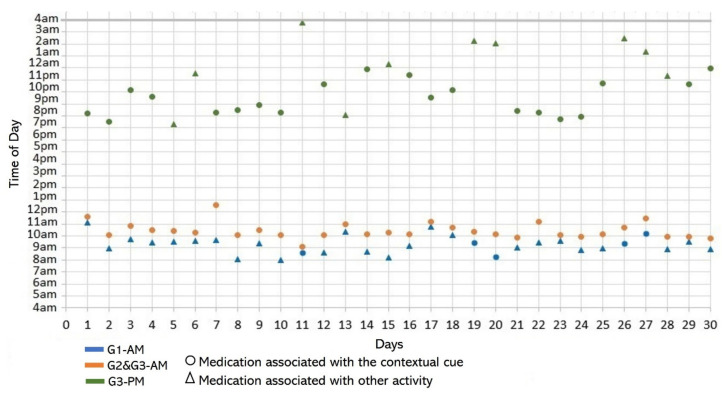
Medication behavior of S3 shows the time of day in which the medication episodes (G1-AM, G2&G3-AM, and G3-PM) were conducted and whether they were associated with the activities set as contextual cues.

**Figure 8:**
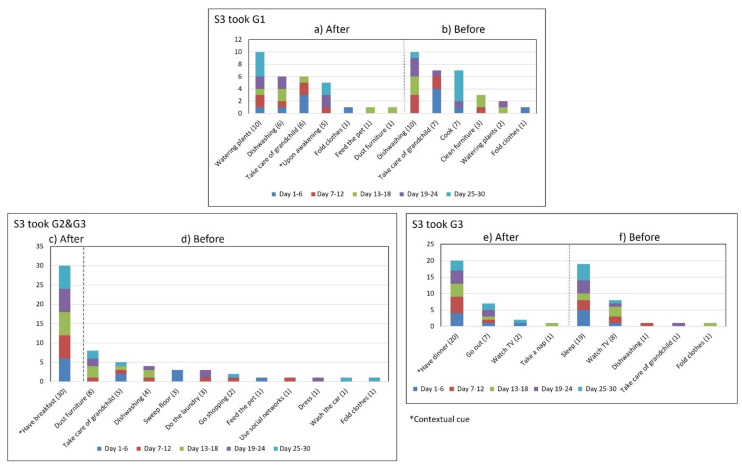
The days that S3 performed their medication episodes (G1-AM, G2&G3-AM, and G3-PM) after and before the reported activities.

**Figure 9:**
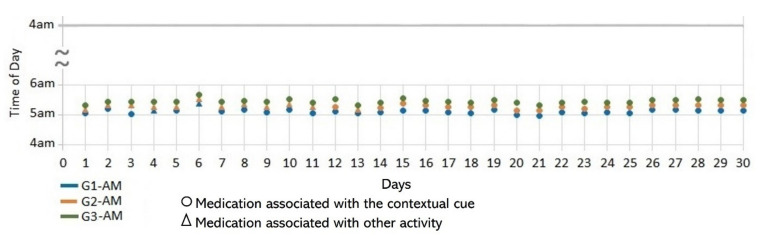
Medication behavior of S4 shows the time of day in which the medication episodes (G1-AM, G2-AM, and G3-AM) were conducted and whether they were associated with the activities set as the contextual cues.

**Figure 10:**
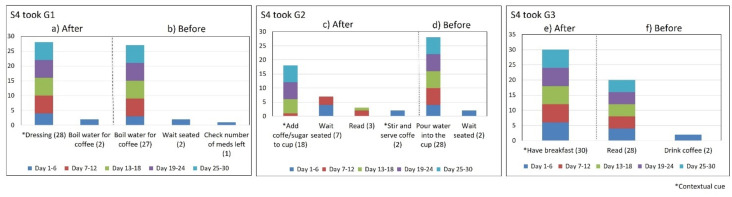
The number of days that S4 performed the medication episodes (G1-AM, G2-AM, and G3-AM) after and before the reported activities.

This section describes the data collected during the initial interview and the 30-day participatory sensing study. We partially monitored the medication regimens of subjects S1 and S4, who needed their children’s assistance when activating the system and therefore only provided data on their morning medication episodes when their children were available to help. Table 1 summarizes the characteristics of the medication episodes that subjects reported daily during the sensing study stage. Figure 2 depicts the location and pills containers used by the subjects. Figure 3, Figure 4, Figure 5, Figure 6, Figure 7, Figure 8, Figure 9 and Figure 10 report the consistency concerning the medication time and activities performed before and after each medication episode, and Table 2 presents the results of the consistency measures. To contextualize the subjects’ medication behaviors, we provide data on their complete medication regimens and routines reported during the semi-structured interviews, as presented in Table 3 for subject 1. The data for all participants are in the Supplementary Material Section (see Tables S1–S4). 

Subject #1 (S1) reported in the initial interview taking one daily dose of two medications that were kept in a container in her bedroom (see Figure 2a): one medication (G1) upon awakening and the other (G2) after breakfast. However, she stopped taking G1 on the 4th day of the study due to instructions from her doctor (see Figure 3). We therefore analyzed her medication behavior regarding G2. Additionally, she reported suffering a fall, which slightly restricted her physical mobility. The results of the participatory sensing data collection showed that she used the cue only 50% of the time (15 days), i.e., taking G2 after having breakfast (see Figure 4). The variances in the *time of day* (*S^2^* = 0.29) and *time interval* (*S^2^* = 0.14) were small.

Subject #2 (S2) reported in the initial interview taking one daily dose of four (G1) different medications after awakening and drinking a smoothie, since the medicine for her diabetes cannot be taken with an empty stomach. She had a pill organizer on the dining room table and another in her handbag to be used if she left the house (see Figure 2b). During 83% of the participatory sensing study days (i.e., 25 days), she took medications when the contextual cue arose, i.e., after drinking a smoothie (see Figure 5 and Figure 6). However, the variances of the *time of day* (*S^2^* = 1.94) and *time interval* (*S^2^* = 3.30) were higher than 1.0.

Subject #3 (S3) reported during the initial interview performing four medication episodes for taking three medications that were kept on a bedside table to make them visible (see Figure 2c). The G1-AM episode included once-daily doses of a medicine that should be taken after awakening on an empty stomach. She took the G3 meds twice a day after meals. She reported taking G2 and G3 after breakfast in the same AM episode and the second doses of G3 after dinner (PM). According to the participatory sensing study, her cues-usage rates for each episode varied: 17% (5 days) for G1-AM, 100% (30 days) for G2-AM and G3-AM, and 66.6% (20 days) for G3-PM (see Figure 7 and Figure 8). The variances in the *time of day* were higher than 1 for the G3-PM episode (*S^2^* = 5.54) in contrast to the variances for the G1-AM episode (*S^2^* = 0.56) and G2&G3-AM episode (*S^2^* = 0.45). Not performing G3-PM consistently at the same time of day incremented the *time interval* variance (*S^2^* = 4.72), while for G1 *(S^2^* = 1.00) and G2 (*S^2^* = 0.93), it was equal to or lower than 1.

Subject #4 (S4) reported taking once-daily doses of six medications that he kept on the dining table (see Figure 2d). To avoid stomach problems, he formed three groups of drugs to be taken 15 min apart. In the initial interview, he reported that the first medication (G1) was taken after getting dressed, the medications of G2 were taken while making coffee, and the medications of G3 were taken after having breakfast. The usage rates of these contextual cues were 93%, 60%, and 100%, respectively (see Figure 9 and Figure 10). The *time of day* and *time interval* variances were low for all of the medication groups (*S*^2^ = 0.01). 

## 4. Discussion 

### 4.1. RQ1: How Does the Association That Older Adults Establish between Their Daily Routines and Their Medication Taking Enable Them to Perform It Consistently?

From the quantitative results, it is difficult to identify which contextual cues are, in general, better than others, since what works for some older adults might not work for everyone. As depicted in Table 2, some medication-taking behaviors that were the most consistent in terms of the time of day were characterized by a high contextual cue consistency, such as the G1-AM of S4 and the G2-AM of S3. However, the constant association of the same activity with medication-taking behavior does not generate greater consistency for all subjects. For example, S1 and S2 performed one medication episode per day. However, S1 shows low cue-usage rates with a high time-based consistency (low variances), while S2 shows a higher cue-usage rate with a lower time-based consistency (high variances). On the other hand, S3 presented a more consistent behavior in the AM episode than in the PM. Using a contextual cue consistently may not be good enough if it is not triggered at the same time of day.

Our results suggest that older adults are not aware of the effect that their selection of contextual cues has on their medication behaviors. Making them aware of their medication behaviors would allow them to realize if they need to adjust or change the daily routines associated with their medication behaviors. The generation of electronic medication daily logs could help provide such awareness if it presents information related to medication behaviors and daily routines.

### 4.2. RQ2: What Problems Do Older Adults Face in Associating Their Daily Routines with Their Medication Taking?

- *Associating broad routines with medication-taking behaviors.* We realize that some activities reported to be used as contextual cues belong to a broad daily routine. For some subjects, using a contextual cue that belongs to a broad routine was a restriction for taking the medication consistently. For instance, the contextual cue of S1 was having breakfast, which was part of a broad routine that included dishwashing. Thus, S1 reported for 6 days that the sequence of her activities was taking breakfast, taking G2 meds, and washing the dishes (see Figure 4). However, for the other 3 days, she took G2 after dishwashing, and on 2 of these days, the medication episodes were performed later than usual (see days 5 and 8 of Figure 3). These results suggest that various activities that comprise a broad routine may generate competition between them and dilute the contextual cue [17]. Therefore, the defined contextual cue lost intensity and could not be perceived. We also noticed that S1 did not habitually conduct this broad routine since she used the contextual cue 50% (15 days) of the time. The lack of evidence on adopting this broad routine as a contextual cue was due to mobility restrictions after her fall.
- *Multiple daily routines are associated with a contextual cue.* Some older adults associated numerous daily routines with a contextual cue. During the initial interview, S3 reported, *“I take the medication [G1] in the morning, after waking up or a little later, because I have to take care of my grandchild, water my plants, and clean the kitchen.”* The data collected during the study shows that she took G1 after waking up for 5 days (see Figure 8) and that she used to carry out different habitual routines, such as watering the plants (10 days), taking care of her grandson (6 days), and cleaning the kitchen (6 days). Unfortunately, associating multiple daily behaviors with the same contextual cue may reduce the probability that any of these behaviors become a habit [17].
- *Lack of strict routines.* S4 had a habitual morning broad routine in which he integrated the medication episodes for taking G1, G2, and G3. They were accomplished consecutively in a short period (see Figure 9). His routine included waking up at 5:00 a.m., dressing, making instant coffee, reading the newspaper, and having breakfast (Figure 10, middle graph). He obtained a time-based consistent behavior for taking all the medications, even though he got a low rate for using “making coffee” as a contextual cue of G2 (66.6%). This is because G2 was associated with different tasks carried out in the middle of the broad routine, in contrast to G1 and G3, which were taken consistently after “dressing” (see Figure 10, left graph) and after “having breakfast” (see Figure 10, right graph) respectively. We conclude that S4′s medication behavior was characterized by a rule-based process he established and followed. It included a sequence of daily activities and medications alternated at specific intervals and carried out in the same place (dining table).

Unlike S4, participants S1 and S3 recognized the lack of a systematic realization of habitual routines. S1 reported not following a strict morning routine after waking up and taking G1: *“...then, If I do not leave the house, I watch television or go out to the garden to water the plants, then I take the next medicine [G2].”* As illustrated in Figure 3, G1 was suspended in the first week of the study. While taking G1 and G2, the time of day of G2 medication episodes varied from one day to another. After discontinuing the intake of G1, she reported adjusting her morning routine: *“...as the doctor asked me to stop taking the medication [G1], I’ve been waking up late ….”* Similarly, S3 identified that a variety of activities carried out characterized specific periods of the day. For example, Figure 8 shows that watering the plants was the routine most (10 days) conducted before the G1-AM medication episode, followed by taking care of her grandchild (6), cleaning the kitchen (6), and waking up (5). We also found that even though S3 consistently performed the AM episode for taking G3, the lack of habitual behavior during the evening caused her to not comply with the interval of time of taking it every 12 h (see Table 2 and Figure 7).

The above-described cases show that older adults with unstructured periods of the day in which they embed their medication reduce the probability of forming medication habits.

- *Disruption of daily routines.* Previous studies demonstrated that older adults recognized that facing unexpected or unplanned activities delays performing medication taking, and they were more likely to forget it [25,27,28]. In contrast to these studies, we found that when older adults plan to engage in activities that disrupt their daily routine, they displace the activity that acts as a contextual cue, thus displacing medication intake. For example, S2 said during the initial interview: *“when I go shopping, I take the morning medication [G1] earlier or later than usual...”.* The data we collected confirmed this behavior, as she reported getting up earlier than usual to go shopping on 4 days (2, 9, 23, and 30, shown in Figure 5). However, on most of these days (9, 23, and 30), she also performed the activity established as a contextual cue before going shopping and then took the medication, which affected its time-based consistency. Similarly, S3 reported carrying out the episode G3-PM with the contextual cue (after dinner) for 20 days (see Figure 8, right). On 7 days of these 20 days, she went out of her home and dined later than usual (days 6, 11, 15, 19, 20, 26, and 27), as shown in Figure 7. On other days she reported spending time doing different activities during the afternoon, e.g., *“[on day 14] I was watching TV, and the time passed…”* and *“[on day 16] I fell asleep, and I didn’t realize the time,”* which led her to have dinner later and to delay taking her medications.

We conclude that older adults who preserved the use of the contextual cue when their daily routines were interrupted were unaware it would affect the consistency of their medication taking. 

### 4.3. Considerations for Designing Habit-Formation Interventions

Following complex and changing medication regimens could lead to unintentional non-adherence to the medication [39]. Three adherence management elements have been identified that are the most successful in coping with unintentional and intentional non-adherence: *measurement, education, and motivation* [24]. Based on our findings and the literature review on habit-forming strategies, we propose a set of recommendations for designing interventions that support these three elements to form medication-taking habits. 

#### 4.3.1. Measuring for Habit Formulation and Awareness Provision 

Electronic measuring has been identified as the main component for improving adherence by a meta-analysis of clinical trials [40]. Electronic measuring alone, without feedback, improves adherence because the patients know they are being monitored [24,41]. However, measuring and feedback improve adherence more than measuring alone [41,42,43]. Thus, this element refers to the fact that older adults need an *awareness* of achieving their medication goals, which can be accomplished through *measurement* [24]. Awareness refers to giving patients feedback on their dosing patterns [24]. 

Previous works have studied the benefits for patients and clinicians of generating observations of daily living (ODLs) through sensing technology [24,44]. Reviewing patients’ ODLs during clinical consultations helps physicians focus on areas that require more attention [44]. It allows them to confirm whether patients are adherent to medication and if it is linked to health outcomes, e.g., if the alterations in a diabetic patient’s glucose level are due to the lack of medication adherence. On the other hand, providing older adults with the digital support to visualize their medication behaviors status (i.e., pending, completed, not completed) enables them to increase their awareness, identify medication errors, and confirm if they took medication, and, based on the above, they can self-regulate their medication-taking behaviors [44]. In contrast to these studies, we have used participatory sensing technology and interviews to understand the individualized constraints of older adults to link their medications with daily activities in order to form medication-taking habits. 

We argue that measuring is essential for providing awareness to patients about medication adherence and enabling clinicians and patients to realize the consistency of the medication behaviors. On the other hand, our results could inspire the development of sensing technologies that allow for the generation of lifelogging systems [45] to monitor how medication behaviors are embedded in patients’ daily activities. We have performed preliminary work in this direction by developing an audio-based activity recognition system that continuously listens to sounds to recognize older adults’ activities through machine learning algorithms [46].

#### 4.3.2. Educational Strategies to Acquire Medication Habits

Traditionally, providing information has been the most used and studied strategy to address the medication adherence problem in older adults, including individualized and group teaching with one or several hours of instruction, written instructions, and home visits [10]. However, to be effective, the educational strategy should complement behavioral strategies to form habits [10]. Vrijens et al. propose that habit formation interventions should provide patients with *knowledge* about the prescribed medication regimens and the importance of adhering to them through *education* [24]. 

Our results show that even though older adults already tend to link their medication-taking behaviors with their routines [20,25], these are not properly selected and used. Based on the above, education for habit formation should also focus on supporting training in the formulated intentions of taking medication linked with specific daily activities. This training can be carried out through reminders delivered by systems easy to configure for this aim, such as mobile phones and smart speakers. Recent studies indicate that older adults are adopting smart speakers since they support an interaction model that does not require experience using computing devices [47]. Older adults mainly use them to cope with memory issues through setting timers and reminders [47]. However, these are passive alerts, so the need to develop intelligent memory and routine virtual assistants has been identified [48]. Designers of habit formation apps should avoid features that teach users to rely on technology, such as using only time-based reminders, which may interfere with developing associations between contextual cues and the medication taking [49].

#### 4.3.3. Motivate through Natural Reinforcements

Patients could improve their self-efficacy for medicating through *motivational* strategies, e.g., providing rewards, such as positive *reinforcements,* that help patients realize that they can reach their medication goals [24]. Positive reinforcement can be a favorable outcome or reward after the desired behavior is performed. It can be a promise or be presented as a possibility of obtaining a good outcome or compensation if an action is performed [50]. The definition of reinforcement is more extensive since it is a consequence applied to strengthen a behavior to make it more likely to be repeated [50]. Psychologists have identified different reinforcers that can be used to reward for performing specific behaviors [51]. They state that, for habit formation, intrinsic motivation, through natural reinforcers, is more effective than extrinsic motivation [12,51].

Studies suggest that healthcare providers must maintain regular contact with patients to reinforce their trust in the benefits they acquire by following their medication-taking habits, including achieving an optimal health outcome [21]. However, in the setting in which our study was conducted (see Section 3.1), older adults did not maintain regular contact with healthcare providers; therefore, they did not assess their medication behaviors with the frequency necessary to reinforce them. In addition, the lack of adequate pharmaceutical policies to rationally manage medications and provide a follow-up to patients’ treatment increases the vulnerability of Mexican seniors to medication errors [52,53]. This confirms the necessity of developing accessible systems by which older adults can form medication-taking habits. Studies suggest the content of reinforcing messages, which habit-forming systems could deliver automatically [46,49].

### 4.4. Limitations of the Study

Even though our study was limited to following only the inductive approach for data codification, carried out by a single author, it was validated through an iterative discussion process conducted in accordance with Braun & Clarke’s guidance [38].

We observed that the participants did not report omitting to take their medications, which might be because our tablet-based system and the economic incentive motivated them to take their medications daily. However, it did not affect the key data we were interested in, and we were still able to assess the use of cues throughout the study period. 

The majority of our participants were women, and we observed that the participant with the best medication-taking behaviors was the male participant (S4), suggesting that female older adults might have more complicated daily routines than male participants due to more responsibilities related to housework. However, because of the small and unbalanced number of participants, we cannot draw strong conclusions regarding the impact of gender. We recognize that including more subjects with different characteristics, such as those living alone or younger adults, would help identify other problems for selecting contextual cues. Additionally, the small sample size and the lack of consideration of other factors such as participants’ psychological distress and the diversity in their dosage patterns could have influenced their consistency of medication-taking behaviors. As such, our results cannot be generalized to different settings and countries, although it should be noted that the results and the types of cues participants selected were in line with prior research, e.g., [28].

Finally, while the overall sample for this study was small, it was suitable for the methods we used, the scope of the study, and the topic investigated. This is because we used the contextual inquiry technique for which at least four subjects are required to understand their tasks [31]. However, we do acknowledge that in order to identify the essential system design requirements based on the data collected in this study, we need to perform further analyses, which is beyond the scope of this manuscript.

## 5. Conclusions

Older adults who matched their medication regimens to their habitual routines obtained better results on time-based consistency measures of medication-taking behaviors. However, they may need assistance to form routines that support more than one medication episode linked with a contextual cue, especially when taking several medications at different interval times (e.g., every 8 h, 12 h), and to change their habits when their prescriptions are modified as their health condition evolves. We identified that the main problems for not using daily routines appropriately as contextual cues were *the insertion of medication-taking behaviors into broad daily routines, the association of multiple daily routines with a contextual cue, the lack of strict daily routines,* and *the disruption of daily routines.* We found that linking drugs with broad activities could work for older adults when these are routinely performed. We argue that the strategies for forming medication-taking habits should support the selection of contextual cues by measuring patients’ dosage patterns and generating logs of their daily activities.

## Figures and Tables

**Table 1:** Characteristics of the participants.

ID	Age	Gender	Diseases	Group of Meds (N)	Episode Time	Contextual Cue
S1	70	Female	Cholesterol, Hypertension	G1 (1)	AM ≈ 8:00	Upon awakening
				G2 (1)	AM ≈ 10:00	After breakfast
S2	72	Female	Hypertension, Diabetes, Gastritis	G1 (4)	AM ≈ 8:30	After drinking a smoothie
S3	72	Female	Hypertension, Diabetes, Osteoporosis, Heart disease	G1 (1)	AM ≈ 8:00	Upon awakening
				G2 (1)	AM ≈ 9:00	After breakfast
				G3 (2)	AM ≈ 9:00	After breakfast
					PM ≈ 19:00	After dinner
S4	73	Male	Hypertension, Diabetes, Thyroid. Angina pectoris	G1 (1)	AM ≈ 5:00	After dressing
				G2 (2)	AM ≈ 5:15	When making coffee
				G3 (3)	AM ≈ 5:30	After breakfast

**Table 2:** Results of the consistency measures.

Subjects	Medication Episodes		Cue Consistency	Time Interval (h)			Time of Day (h)		
				*M* ^d^	*SD* ^e^	*S^2^* ^f^	*M* ^d^	*SD* ^e^	*S^2^* ^f^
S1	G2 ^a^-AM		50%	23.97	0.37	0.14	10.39	0.54	0.29
S2	G1 ^a^-AM		83%	23.86	1.82	3.30	8.90	1.39	1.94
S3	G1 ^a^-AM		17%	23.92	1.00	1.00	9.28	0.75	0.56
	G2 ^a^-AM ^c^		100%	23.94	0.97	0.93	10.43 ^a^	0.67 ^a^	0.45 ^a^
	G3 ^b^-	AM^c^		11.90 ^b^	2.17 ^b^	4.72 ^b^			
		PM	66.6%				22.38	2.35	5.54
S4	G1 ^a^-AM		93%	24.00	0.10	0.01	5.12	0.08	0.01
	G2 ^a^-AM		60%	24.01	0.10	0.01	5.28	0.07	0.01
	G3 ^a^-AM		100%	24.01	0.10	0.01	5.45	0.07	0.01

^a.^ Group of medications taken every 24 h. ^b.^ Group of medications taken every 12 h. ^c.^ Groups of medications taken in the same episode (i.e., simultaneously). ^d.^ Mean. ^e.^ Standard deviation. ^f.^ Variance.

**Table 3:** Demographic and medication routine characteristics gathered from subject S1 during the initial interview.

Subject	ID	Gender		Age (Years)		Living with:	
	S1	Female		70		Her Daughter and Grandchildren	
**Medication characteristics**	**Health problem**	**Prescription** **^a,c^**			**Reported cues used to take the medication**		
		**Medication**	**Doses (pills)**	**Daily frequency**	**Time**	**Associated activity**	**^b^** **Med episodes**
	Cholesterol	Pravastatin	1	24 h	≈8:00	Upon awakening	G1-AM
	Hypertension	Amlodipine	1	24 h	≈10:00–10:30	After breakfast	G2-AM
	Fluid retention	Chlortalidone	1	24 h	≈12:00	Watering plants	n/m
	Pain	Indomethacin	1	24 h	≈14:00	Before watching favorite TV-show	n/m
	Pain	Tramadol	1	24 h	≈16:00	Before watching favorite soup opera	n/m
	Depression	Mirtazapine	1	24 h	≈20:00–22:00	Before sleeping	n/m
**Routine description**	“I kept notes of the time I took the medication for a long time until I learned how to do it, and I don’t forget to take the pills. I have two pill boxes, a weekly one and a smaller one [with one compartment] to store the pills to take during the day. Sunday, I go to the weekly pill box, separate the pills, and add them to the seven compartments of the pill box [one for each day]. Every night I put the pills for the next day into the small pillbox [with one compartment]; I distinguish the pills by their size and color… As soon as I wake up, I get up and take the pravastatin that controls the cholesterol. I have the pill box on the nightstand in the bedroom, near a glass of water. Then, I go to the kitchen, make coffee, eat some toast, go back to my room, make the bed, clean the room a bit, and sometimes watch TV. Between 10:00 a.m. and 10:30 a.m., I have breakfast and take the amlodipine pill to control blood pressure. If I don’t leave the house, I watch television or go out to the patio to water the plants. I take the following medicine, chlorthalidone, right away, I have lunch, and I start to watch my favorite program, which is at 2:00 p.m., just when I take the next drug, the indomethacin. At 4:00 p.m., the soap opera that I like starts, which indicates me to take the following drug, tramadol. After 5:00 p.m., I take a nap, and between 8:00 p.m. and 10:00 p.m., I take the last medicine before I go to sleep.”						

n/m: These medication episodes were not monitored during the sensing study. ^a.^ Information obtained from the written prescription provided by subject’s doctor; ^b^ Medication episodes that were monitored during the sensing study; ^c.^ For this subject, there are no medical instructions to take the medications when performing specific activities, e.g., after eating.

## Data Availability

The data presented in this study are available in this article and the Supplementary Material section.

## Supplementary Materials

The following supporting information can be downloaded at: https://www.mdpi.com/article/10.3390/healthcare10071238/s1. Table S1. Demographic and medication routine characteristics gathered from subject S1 during the initial interview; Table S2. Demographic and medication routine characteristics gathered from subject S2 during the initial interview; Table S3. Demographic and medication routine characteristics gathered from subject S3 during the initial interview; Table S4. Demographic and medication routine characteristics gathered from subject S4 during the initial interview.
