# Supplementary material for: A Participatory Sensing Study to Understand the Problems Older Adults Faced in Developing Medication-Taking Habits
Source: Healthcare (Basel). 2022 Jul 2;10(7):1238. doi: 10.3390/healthcare10071238 (PMC9323283; doi:10.3390/healthcare10071238)
Supplement: Supplementary file 1 [file healthcare-10-01238-s001.zip › healthcare-1739109-supplementary.pdf]

Table S1. Demographic and medication routine characteristics gathered from subject S1 during the initial interview.

Subject	ID	Gender	Age (years)	Living with:
	S1	Female	70	Her daughter and grandchildren

Medication characteristics	Health problem	Prescription[a,c]			Reported cues used to take the medication		
		Medication	Doses (pills)	Daily frequency	Time	Associated activity	[b]Med episodes
	Cholesterol	Pravastatin	1	24 hrs	≈ 8:00	Upon awakening	G1-AM
	Hypertension	Amlodipine	1	24 hrs	≈ 10:00 – 10:30	After breakfast	G2-AM
	Fluid retention	Chlortalidone	1	24 hrs	≈12:00	Watering plants	n/m
	Pain	Indomethacin	1	24 hrs	≈14:00	Before watching favorite TV-show	n/m
	Pain	Tramadol	1	24 hrs	≈16:00	Before watching favorite soup opera	n/m
	Depression	Mirtazapine	1	24 hrs	≈ 20:00-22:00	Before sleeping	n/m

Routine description

"I kept notes of the time I took the medication for a long time until I learned how to do it, and I don't forget to take the pills. I have two pill boxes, a weekly one and a smaller one [with one compartment], to store the pills to take during the day. Sunday, I go to the weekly pill box, separate the pills, and add them to the seven compartments of the pill box [one for each day]. Every night I put the pills for the next day into the small pillbox [with one compartment]; I distinguish the pills by their size and color… As soon as I wake up, I get up and take the pravastatin that controls the cholesterol. I have the pill box on the nightstand in the bedroom, near a glass of water. Then, I go to the kitchen, make coffee, eat some toast, go back to my room, make the bed, clean the room a bit, and sometimes watch TV. Between 10:00 am and 10:30 am, I have breakfast and take the amlodipine pill to control blood pressure. If I don't leave the house, I watch television or go out to the patio to water the plants. I take the following medicine, chlorthalidone, right away, I have lunch, and I start to watch my favorite program, which is at 2:00 pm, just when I take the next drug, the indomethacin. At 4:00 pm, the soap opera that I like starts, which indicates me to take the following drug, tramadol. After 5:00 pm, I take a nap, and between 8:00 pm and 10:00 pm, I take the last medicine before I go to sleep."

n/m: These medication episodes were not monitored during the sensing study.
a. Information obtained from the written prescription provided by subject's doctor
b Medication episodes that were monitored during the sensing study.
c. For this subject, there are no medical instructions to take the medications when performing specific activities, e.g., after eating.

Table S2. Demographic and medication routine characteristics gathered from subject S2 during the initial interview.

Subject	ID	Gender	Age (years)	Living with:
	S2	Female	72	Her husband

Medication characteristics	Health problem	Prescription[a]			Additional medical instructions	Reported cues used to take the medication		
		Medication	Doses (pills)	Time interval		Time	Associated activity	Med episodes[b]
	Hyperten-sion, Diabetes, Gastritis	Losartan	12	24 hrs	-	≈ 8:00	After drinking a smoothie	G1-AM
		Chorthali-done	1	24 hrs	-			
		Metformin	1	24 hrs	after eating			
		Ranitidine	1	24 hrs	-			

Routine description	
	"I don't have a schedule to take the medicines. Most of the time, I get up, eat a smoothie, take all four medications, shower, and regularly go to the church at 8:00 am. Sometimes, I take my meds until I get home, at 10:00 am, after eating something. A while ago, I tried to take the meds in multiple episodes, taking my diabetes med in the afternoon and my blood pressure med in the morning; but I often forgot to take the ones for diabetes. I couldn't get used to it. It's better to take them all together...."

a. Information obtained from the written prescription provided by subject's doctor
b Medication episodes that were monitored during the sensing study.

Table S3. Demographic and medication routine characteristics gathered from subject S3 during the initial interview.

<table>
<tr><th rowspan="2">Subject</th><th>ID</th><th colspan="3">Gender</th><th colspan="2">Age (years)</th><th colspan="2">Living with:</th></tr>
<tr><td>S3</td><td colspan="3">Female</td><td colspan="2">72</td><td colspan="2">Her husband</td></tr>
<tr><th rowspan="6">Medication characteristics</th><th rowspan="2">Health problem</th><th colspan="3">[a]Prescription</th><th rowspan="2">Additional medical instructions</th><th colspan="3">Reported cues used to take the medication</th></tr>
<tr><th>Medication</th><th>Doses (pills)</th><th>Time interval</th><th>Time</th><th>Associated activity</th><th>Med episodes</th></tr>
<tr><td rowspan="4">Osteoporo-sis, Diabetes, Heart disease</td><td>Alendronic acid</td><td>1</td><td>24 hrs</td><td>on an empty stomach</td><td>≈ 8:00</td><td>Upon awakening</td><td>G1-AM</td></tr>
<tr><td>Caltrate</td><td>1</td><td>24 hrs</td><td>-</td><td>9:00</td><td>after breakfast</td><td>G2-AM</td></tr>
<tr><td>Metformin</td><td>1</td><td>12 hrs</td><td>after eating</td><td>≈ 9:00 & ≈19:00</td><td>after breakfast & after dinner</td><td rowspan="2">G3-AM & G3-PM</td></tr>
<tr><td>Enalapril</td><td>1</td><td>12 hrs</td><td>-</td><td>≈ 9:00 & 19:00</td><td>after breakfast & after dinner</td></tr>
<tr><th>Routine description</th><td colspan="8">“I take alendronic acid on an empty stomach. Sometimes I take it when I wake up, and other times later, after sweeping the street and patio or watering the plants. After breakfast, I take caltrate, metformin, and enalapril….. I take the ones in the afternoon after finishing my activities. Sometimes I take them before going to bed or when I'm watching TV. I am careful that 12 hours elapse between taking these drugs [metformin, enalapril]. At night I am not in the habit of having a formal dinner, but I try to eat something and take my medication… I always have my medications at home, but if I go out, I carry them in my bag. I always have a box with the medicines in my bag, so I don't forget to take them when I go out. Thus, when I go to Calexico, CA, to visit my sister, I carry the medications in my bag to take them wherever I am. If I have an appointment with the doctor, I also bring my meds with me, and until I'm free and have breakfast, I take them.”</td></tr>
<tr><td colspan="9">a. Information obtained from the written prescription provided by subject’s doctor
b. Medication episodes that were monitored during the sensing study.</td></tr>
</table>

Table S4. Demographic and medication routine characteristics gathered from subject S4 during the initial interview.

Subject	ID	Gender	Age (years)	Living with:
	S4	Male	73	Her wife and daughter

Medication characteristics

Health problem	Prescription			Additional medical instructions	Reported cues used to take the medication		
	Medication	Doses (pills)	Time interval		Time	Associated activity	
Thyroid	Levothyro-xine	1	24 hrs	on an empty stomach	≈ 5:00	after dressing	G1-AM
Diabetes	Metformin	1	24 hrs	-	≈ 5:15	when making coffee	G2-AM
Blood coagulation	Aspirin	1/4	24 hrs	-			
Angina pectoris	Isosorbide	1	12 hrs	after eating	≈ 5:30 & ≈ 19:00	after breakfast & after a snack	G3-AM
Blood pressure	Losartan	1	12 hrs	-	≈ 5:30 & ≈ 19:00	after breakfast & after a snack	
Blood pressure	Metoprolol	1	12 hrs	-	≈ 5:30 & ≈ 19:00	after breakfast & after a snack	

Routine description

I take the pill for my thyroid at 5:00 am, when I wake up and on an empty stomach. Then I take my diabetes pill and aspirin, so I don't have a heart attack. I don't take all the medications together [when I wake up] because isosorbide makes me nauseous if I take it on an empty stomach. I have to take it after I eat something.... At 7:00 - 8:00 pm, I take my blood pressure and heart meds; I eat a snack and then take these meds. If my chest hurts, I put a pill under my tongue. If the pain doesn't disappear, I must go to the hospital immediately.

a. Information obtained from the written prescription provided by subject’s doctor
b Medication episodes that were monitored during the sensing study.
